# Prevalence of type 2 diabetes in Nepal: a systematic review and meta-analysis from 2000 to 2014

**DOI:** 10.3402/gha.v8.29088

**Published:** 2015-11-26

**Authors:** Bishal Gyawali, Rajan Sharma, Dinesh Neupane, Shiva Raj Mishra, Edwin van Teijlingen, Per Kallestrup

**Affiliations:** 1 Center for Global Health, Department of Public Health, Aarhus University, Aarhus, Denmark; 2 Nepal Development Society (NEDS), Bharatpur, Nepal; 3 Faculty of Health & Social Sciences, Bournemouth University, Dorset, United Kingdom

**Keywords:** type 2 diabetes, systematic review, prevalence, epidemiology, South Asia

## Abstract

**Background:**

Understanding the prevalence of type 2 diabetes in Nepal can help in planning for health services and recognising risk factors. This review aims to systematically identify and collate studies describing the prevalence of type 2 diabetes, to summarise the findings, and to explore selected factors that may influence prevalence estimates.

**Design:**

This systematic review was conducted in adherence to the MOOSE Guidelines for Meta-Analysis and Systematic Reviews of Observational Studies. Medical Literature Analysis and Retrieval System (MEDLINE) database from 1 January 2000 to 31 December 2014 was searched for the prevalence of type 2 diabetes among Nepalese populations with a combination of search terms. We exploded the search terms to include all possible synonyms and spellings obtained in the search strategy. Additionally, we performed a manual search for other articles and references of published articles.

**Results:**

We found 65 articles; 10 studies fulfilled the inclusion criteria and were included in the analyses. These 10 studies comprised a total of 30,218 subjects. The sample size ranged from 489 to 14,009. All the studies used participants older than age 15, of whom 41.5% were male and 58.5% female. All the studies were cross-sectional and two were hospital-based. Prevalence of type 2 diabetes ranged from a minimum of 1.4% to a maximum of 19.0% and pooled prevalence of type 2 diabetes was 8.4% (95% CI: 6.2–10.5%). Prevalence of type 2 diabetes in urban and rural populations was 8.1% (95% CI: 7.3–8.9%) and 1.0% (95% CI: 0.7–1.3%), respectively.

**Conclusions:**

This is, to our knowledge, the first study to systematically evaluate the literature of prevalence of type 2 diabetes in Nepal. Results showed that type 2 diabetes is currently a high-burden disease in Nepal, suggesting a possible area to deliberately expand preventive interventions as well as efforts to control the disease.

Approximately 387 million people are living with diabetes worldwide with an estimated prevalence of 8.3% in 2014 and is predicted to increase to 10% by 2030 (1). Diabetes caused 4.9 million deaths in 2014, costing 612 billion dollars in health care (1). More than 80% of diabetes deaths are reported in low- and middle-income countries (LMICs) (2). Of the two forms of diabetes, type 2 diabetes is widespread globally, accounting for over 90% of all diabetes cases (2). A systematic review carried out in 2012 confirmed a rapid increase in prevalence over the last two decades in the South Asian region (3). The prevalence of type 2 diabetes in South Asia in 2011, according to the International Diabetes Federation (IDF), is shown in Fig. 1 (4). Factors such as family history, urban residence, advanced age, higher Body Mass Index (BMI), poor lifestyle, and hypertension were found to be major drivers behind the increasing prevalence of diabetes in South Asia (3).

**Fig. 1:**
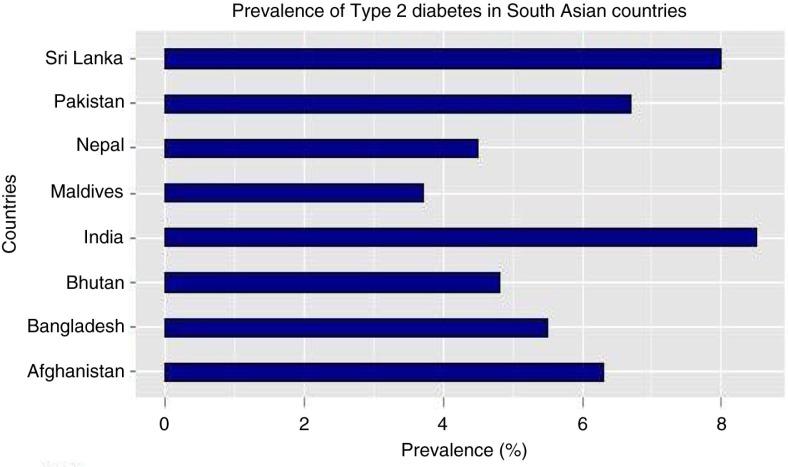
Prevalence of type 2 diabetes in South Asia. Source: International Diabetes Federation, 2012.

Nepal is passing through a phase of epidemiological transition from a higher prevalence of communicable diseases to that of non-communicable diseases (NCDs) and is currently suffering from a double burden of diseases (5). The prevalence of NCDs including type 2 diabetes is expected to increase rapidly in the near future (6). There is a lack of reliable and representative data on the prevalence of type 2 diabetes in Nepal. Various small studies from different parts of the country carried out on the diverse populations have shown varying prevalence rates ranging from 6.3 to 8.5% (7, 8). Individually these studies cannot provide sufficient information about the overall prevalence of type 2 diabetes in the country due to the non-representativeness of the study populations. It is anticipated that bringing together the currently available evidence on the prevalence of type 2 diabetes in Nepal will improve statistical power and provide more accurate estimations to inform policy makers at the local and national level to control the emerging burden of the disease. Thus, the purpose of this review is to document the studies estimating the prevalence of and associated risk factors of type 2 diabetes in Nepal through a systematic review and meta-analysis.

## Methods

### Data selection

This systematic review was conducted in adherence to the MOOSE Guidelines for Meta-Analysis and Systematic Reviews of Observational Studies (9). Data on the prevalence of diabetes among the Nepalese adults were obtained by a three-stage process. In the first stage, a search of the online Medical Literature Analysis and Retrieval System (MEDLINE) database was performed with a combination of Medical Subject Headings (MeSH) terms: ‘Diabetes Mellitus’ as the MeSH major topic and ‘Epidemiology/EP’ as the MeSH subheading. We explored the search terms to include all possible synonyms and spellings obtained in the search strategy. We did not use pre-diabetes as a MeSH term since this search term was only introduced in 2002 and definitions of pre-diabetes cannot be found from studies predating 2002. The search limits were language (‘English’), dates (between ‘1st January 2000’ and ‘31st December 2014’), and species (‘Human’). In addition, the above results were narrowed down by adding ‘Nepal’ as a key word. Two researchers (BG, RS) independently searched the database with these search terms to ensure that none of the relevant studies were missed. Additionally, we performed a manual search for other articles and references of published articles. We identified a number of studies from World Health Organization (WHO) publications (one study) (10), grey literature (two studies) (11, 12), university institutional website (one study) (13), and reference lists of retrieved articles (three studies) (14–16). We attempted to retrieve grey literature through Google. One email request was also sent to a corresponding author to obtain raw data (15), but the attempt was unsuccessful. Since the reported prevalence rate was unusually high, the article was excluded from the analysis, which we believe was the appropriate action.

In the second stage, the total hits obtained from MEDLINE using the above criteria were screened by reading titles and abstracts. Studies not satisfying the inclusion criteria were excluded. The studies identified for inclusion in the second stage were further screened for suitability during the third stage by reading the selected manuscripts. As a part of our search strategy, we included studies only if they met the following criteria: 1) population-based cross-sectional studies reporting the prevalence of type 2 diabetes and published in English; 2) the studies were conducted in Nepal after 2000 and published between 1 January 2000 and 31 December 2014; 3) the study subjects being 15 years and above, including both men and women and not restricted to a specific profession; 4) with information on the survey year, population characteristics, age group, sample size of both men and women, survey procedure, and diagnostic criteria; 5) studies consisting primary data; and 6) studies that defined type 2 diabetes using the WHO, American Diabetes Association (ADA), or both. When there was more than one report relating to the same study sample, the most up-to-date and relevant study was included. All studies were original and contained a minimum of information necessary to calculate pooled analysis of prevalence (number of subjects and number of diabetes events). The full text of studies meeting inclusion criteria was retrieved and screened to determine eligibility by two reviewers (BG, RS).

### Data extraction

For each eligible study, we extracted the following data by means of a structured data extraction form: first author, year of publication, study design, sampling method, urban/rural residence, net sample size (number of included men and women separately), age range, prevalence of pre-diabetes and diabetes, diagnostic method, diagnostic criteria, and reagent used. Where available, odds ratios (ORs) with respective confidence interval (CI) for associated risk factors (gender, age, BMI, family history, physical activity levels, the area of residence, and hypertension) were recorded.

**Fig. 2:**
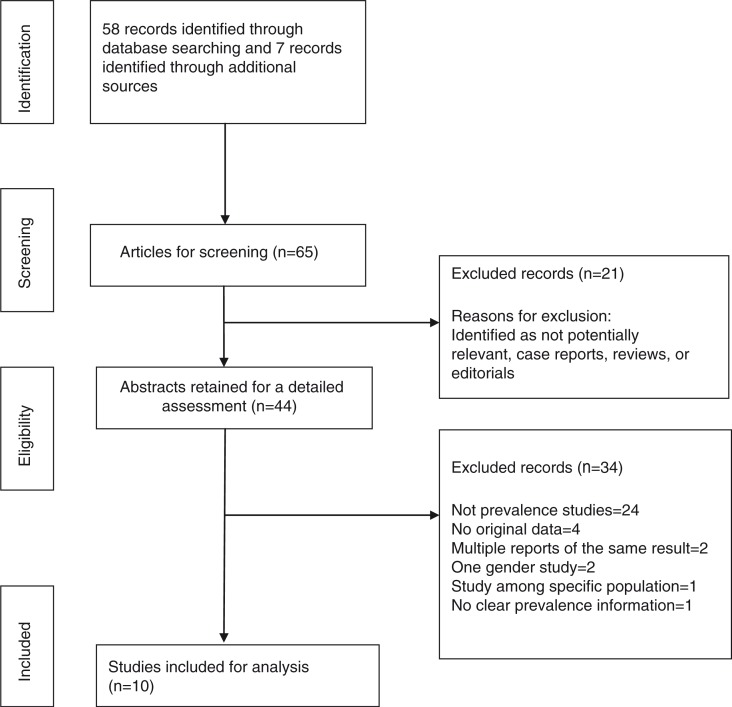
Flow diagram of study.

We entered data in a pre-tested Microsoft Office Excel spreadsheet designed based on the Strengthening the Reporting of Observational Studies in Epidemiology Statement (STROBE) checklist (17). We performed quality assessment of included studies to determine the potential for selection bias based on the presence of eligibility criteria, sampling strategy, sample size, non-response rate, explaining limitations of the study, and generalisability as well as for measurement bias which included measurement techniques (18). These two biases are important in cross-sectional studies that aim to estimate prevalence. A total of 11 domains were assessed. A score of one was given for fulfilling conditions in each domain, 0.5 for partial fulfilment, and 0 otherwise. The maximum possible score was 11 and a study scoring seven or more was classified as a high-quality study and low-quality study otherwise. Each study was rated and the quality of the study has been included in the ‘study characteristics’ table (Table 1). Three studies fulfilled the highest quality criteria (10, 23, 25), while the majority of the articles did not include the limitations of the studies. The detailed inclusion and exclusion criteria as well as extraction process of the articles are shown in Fig. 2.

**Table 1:** Study characteristics

							Prevalence					
											
Study ID	Year of publication	Study design	Sampling method	Sample size (male/female)	Urban/rural	Age group	Pre-diabetes	Type 2 diabetes	Diagnostic method	Diagnostic criteria	Reagent used	Quality
Baral N (19)	2000	Hospital-based cross-sectional	NR*	920 (487/433)	Urban	30–65	9.5	5.2	FPG/OGTT	WHO 1998	Glucose oxidase/peroxidase	Low
Karki P (20)	2000	Hospital-based cross-sectional	NR	1,840 (1,040/800)	Urban	>30	NR	6.3	FPG/OGTT	WHO 1985	Glucose oxidase/peroxidase	Low
Singh DL (21)	2003	Cross-sectional	NR	1,841 (856/985)	Urban and rural	≥20	9.1	14.6	FPG	WHO 1998, ADA 1997	NR	Low
Sasaki H (22)	2004	Cross-sectional	NR	489 (161/328)	Semiurban and rural	>20	2.5	1.4	OGTT	WHO 1998, ADA 1997	Glucose oxidase/peroxidase	Low
Shrestha UK (23)	2006	Cross-sectional	Cluster	1,012 (423/589)	Urban	≥40	11.5	19.0	FPG/OGTT	WHO 1998, ADA 1997	Glucose oxidase/peroxidase	High
Ono K (24)	2007	Cross-sectional	NR	740 (286/454)	Semiurban	>20	19.2	9.5	FPG	NR	NR	Low
Mehta KD (25)	2011	Cross-sectional	Simple random	2,006 (1,096/910)	Urban and rural	≥30	16.9	11.9	FPG	WHO 1998	Glucose oxidase/peroxidase	High
Sharma SK (8)	2011	Cross-sectional	NR	14,009 (5,327/8,682)	Rural	≥20	NR	6.3	FPG	IDF 2009	Glucose oxidase/peroxidase	Low
Sharma SK (26)	2013	Cross-sectional	NR	3,218 (1,542/1,676)	Rural	≥20	NR	7.5	FPG/Urine	ADA 2003	Glucose oxidase/peroxidase	Low
Aryal KK (10)	2013	Cross-sectional	Probability proportionate to size	4,143 (1,336/2,807)	Urban and rural	15–69	4.1	3.6	FPG	WHO 2005	Glucose oxidase/peroxidase	High

* NR=not recorded.

### Study characteristics

Ten studies that fulfilled the inclusion criteria were used for the review (8, 10, 19–26). Publication years ranged from 2000 to 2014. These 10 cross-sectional studies comprised a total sample size of 30,218. The sample size ranged from 489 to 14,009. All the studies used participants older than age 15, comprising 41.5% males and 58.5% females. All of the studies were cross-sectional and two were hospital-based (19, 20). Three studies reported their sampling methods (10, 23, 25): one used simple random sampling (25), one used cluster sampling (23), and the third used probability proportionate to size (10). Three studies reported response rates (10, 23, 25). Five studies reported using fasting plasma glucose (FPG) as the diagnostic method of type 2 diabetes (8, 10, 21, 24, 25): three studies reported both FPG and oral glucose tolerance test (OGTT) (19, 20, 23), one used FPG/urine (26), and one reported OGTT (22). All studies used glucose oxidase/peroxidase for the determination of free glucose in body fluids. Three studies classified urban and rural prevalence of diabetes separately (10, 21, 25). The reported prevalence rates in urban areas were 19.4, 14.6, and 1.4% and in rural areas were 1.3, 2.5, and 0.03%. Studies did not present prevalence rates based on gender, age, and other factors. We could only gather ORs based on those factors. Information regarding prevalence rates based on residence (rural/urban) was present in only three of the studies and we concluded that this stratification would provide some information regarding the distribution of type 2 diabetes. However, the table has not been included in this article. One study used the 1985 WHO diagnostic criteria for diabetes which included the cut-off point for FPG as greater than 7.8 mmol/L (19). The characteristic of studies included in this review is shown in Table 1.

The diagnostic criteria for the presence of ‘type 2 diabetes mellitus’ used in eligible studies were the 1985 criteria from WHO (27), the 1999 WHO criteria (28), the 1997 criteria from the ADA (28), or its updates (29, 30). ‘Pre-diabetes’ was defined as the presence of impaired fasting glucose (IFG) or impaired glucose tolerance (IGT) based on FPG level 6.1–6.9 mmol/l (110–125 mg/dl) or fasting glucose measurement less than 7.0 mmol/l (up to 126 mg/dl). ‘Diabetes mellitus’ was defined based on fasting glucose level greater or equal to 7.0 mmol/l (126 mg/dl) or greater. Values for diagnosis of type 2 diabetes by WHO and ADA are shown in Table 2.

**Table 2:** Values for diagnosis of type 2 diabetes by WHO and ADA

Glucose concentrations, mmol/L (mg/dL)			

	Whole blood		Plasma
		
	Venous	Capillary	Venous
Type 2 diabetes (WHO 1985)			
Fasting	>6.1 (>110)	>6.1 (>110)	>7.8 (>140)
2-hour post-glucose load or both	>10.0 (>180)	>11.1 (>200)	>11.1 (>200)
Type 2 diabetes (ADA 1997, 2003; WHO 1999)			
Fasting	>6.1 (>110)	>6.1 (>110)	>7.0 (>126)
2-hour post-glucose load or both	>10.0 (>180)	>11.1 (>200)	>11.1 (>200)

### Statistical analysis

We performed meta-analysis using STATA version 12 (StataCorp, College Station, TX, USA). We calculated pooled prevalence estimates and 95% CIs by meta-analysis. The pooled prevalence was calculated by using standard error of prevalence that is given by √[*p*×(1−*p*)/*n*], where *p* is the proportion of prevalence and *n* is the reported sample size. Heterogeneity among studies was assessed with the Cochran chi-square (χ^2^) and quantified with the *I*^2^ and tau-square (τ^2^) and the values have been mentioned in the text (31). *I*^2^ is the proportion of total variation provided by between-study variation, and *I*^2^ values of 0, 25, 50, and 75% represent no, low, moderate, and high heterogeneity, respectively (32). τ^2^ is a method of moment estimate of between-study variance (33). The rural and urban categories were made based on the information provided by the individual studies. We evaluated potential risk factors for the prevalence of diabetes by meta-analysis. We used two formal tests: Begg's adjusted-rank correlation test (34) and Egger's regression asymmetry test (35) to evaluate publication bias quantitatively.

Random effects model was carried out to address heterogeneity in the pooled proportions (*P*<0.05 from χ^2^ test) (36). The random effects model assumes that the observed heterogeneity is determined by real differences in the distribution. The model calculated pooled prevalence and 95% CIs. A low *P* value or large chi-squared statistic (relative to its degree of freedom) suggests heterogeneity and variation in effect estimates beyond chance. A two-tailed *P* value <0.05 was considered statistically significant in all analyses.

## Ethical consideration

This article is based on published data, and hence ethical approval is not required.

## Results

### Prevalence of type 2 diabetes

**Fig. 3:**
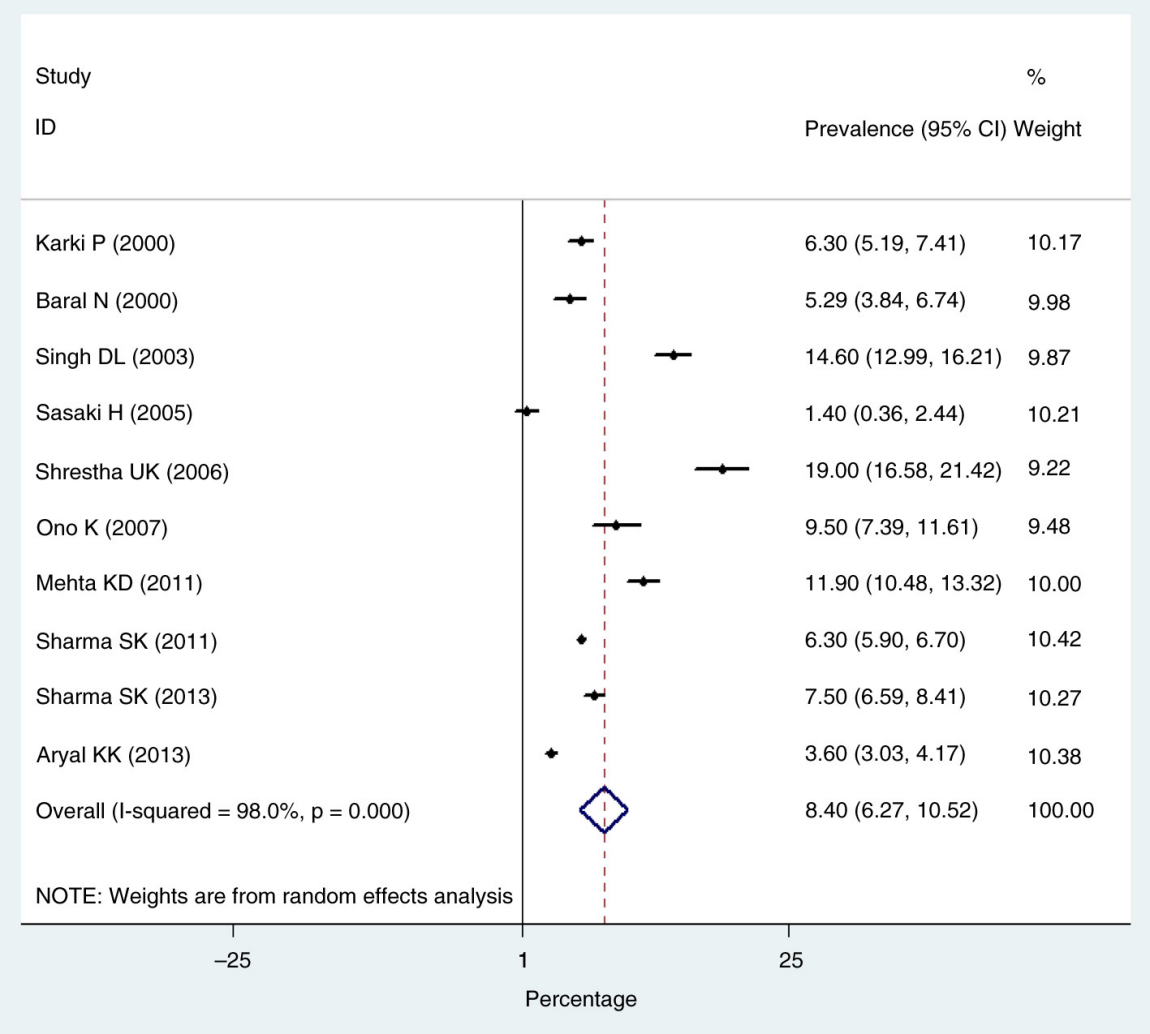
Prevalence of type 2 diabetes in Nepal.

The estimated pooled prevalence of type 2 diabetes in Nepal was 8.4% (95% CI: 6.2–10.5%). We observed a high degree of heterogeneity (*I*^2^=98.0%; τ^2^=11.26; *P*<0.001), with prevalence rates ranging from 1.4 to 19.0%. Figure 3 represents a meta-analysis of studies that measured the prevalence of type 2 diabetes in Nepal. Significant differences in type 2 diabetes prevalence were observed between urban and rural parts of Nepal. The pooled urban prevalence of type 2 diabetes was 8.1% (95% CI: 7.3–8.9%; *I*^2^=99.5%; *P*<0.001) and rural prevalence of type 2 diabetes was 1.0% (95% CI: 0.7–1.3%; *I*^2^=94.2%; *P*<0.001). Seven studies reported the prevalence of pre-diabetes (10, 19–23, 26). The overall prevalence of pre-diabetes was found to be 10.3% (95% CI: 6.1–14.4%; τ^2^=30.43, *I* ^2^=98.4%; *P*<0.001).

### Risk factors of type 2 diabetes

Meta-analysis was performed and pooled ORs were calculated from the adjusted ORs and 95% CI in each available study. The pooled OR from random effect analysis showed that the likelihood of having type 2 diabetes was higher among females (OR=1.6; 95% CI: 1.3–1.9; *I*^2^=87.2%, *P*<0.05) (8, 10, 21, 23, 25, 26). We found pooled OR for increasing age to be 1.05 (95% CI: 1.04–1.05; *I*^2^=48.0%; *P*>0.05) (8, 21, 23, 26) and pooled OR for hypertension to be 2.16 (95% CI: 1.5–2.8: *I*^2^=0.0%; *P*>0.05) (23, 25). One study reported urban residency, having a higher socio-economic status and a higher BMI as risk factors for diabetes (25). One study reported physical activity and primary education as risk factors for diabetes (8). We could not conduct a meta-analysis and calculate pooled ORs of these studies since only one study reported the same risk factor.

### Publication bias

**Fig. 4:**
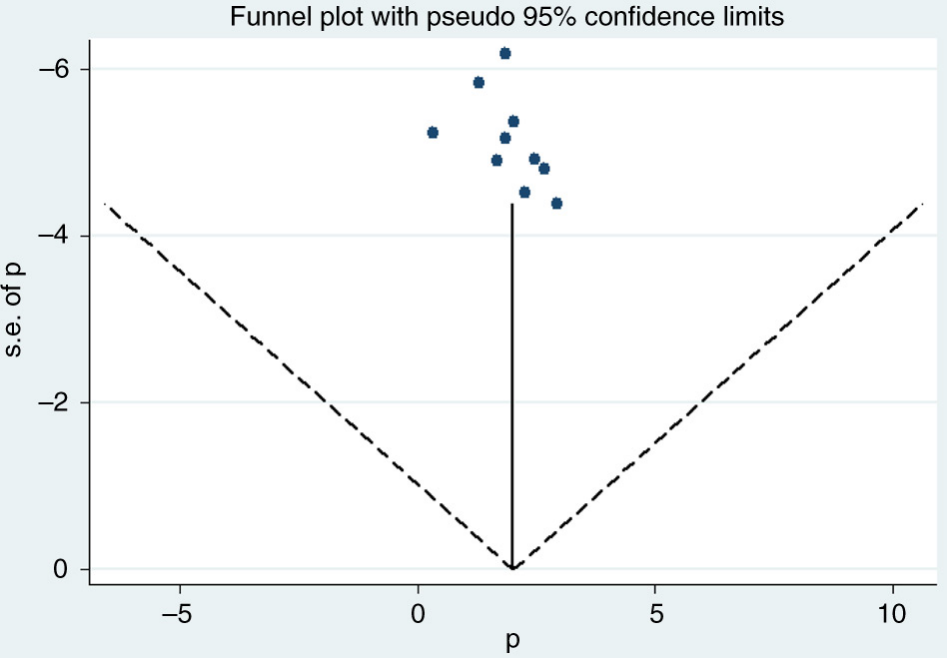
Funnel plot with 95% confidence limits showing the prevalence (*p*) of type 2 diabetes in each study by the standard error (s.e.) of the studies.

The results of the statistical test for publication bias, including Begg's adjusted-rank correlation test and Egger's regression asymmetry test were statistically insignificant (*P*=0.060 and *P*=0.086, respectively). These results showed no evidence of publication bias. Even if these tests are negative, there is still a possibility of publication bias. Figure 4 represents the funnel plot for visualising publication bias amongst the 10 studies used for meta-analysis.

## Discussion

This is the first study, to our knowledge, to systematically evaluate the literature of prevalence of type 2 diabetes in Nepal. This study summarised prevalence of type 2 diabetes in Nepal for a 14-year period (2000–2014). The pooled prevalence of type 2 diabetes was found to be 8.4% (95% CI: 6.2–10.5%) – higher than the current national estimate of 4.5% (4). However, this pooled result is consistent with other literature examining the prevalence of type 2 diabetes in different parts of the world. For instance, prevalence based on the most recent national surveys in neighbouring countries of Nepal that have similar culture and lifestyle profiles (37) were 8.5% in India, 6.7% in Pakistan, and 8% in Sri Lanka according to IDF estimates 2012 (4). Moreover, our study reported the estimates ranged from 1.4 up to 19.0%. This is comparable to the wide range of prevalence rates obtained from a review by Siddiqui and colleagues, which showed prevalence rates of diabetes varying between 0.9 and 21.2% in Bangladesh and India, respectively (38). A Mexican study reported a prevalence of diabetes ranging from around 3 to 20% (39). Various socio-economic characteristics and lifestyle behaviours of rural and urban regions may have attributed to these observed differences between regions. The reported high prevalence of diabetes exemplifies the shift from a burden of disease ruled by mortality from infectious causes to chronic ones (40). This increase could be attributed to various lifestyle changes associated with urbanisation and deterioration of the ecological environment (41). Linking to increase in obesity and reduction in physical activities in recent years in Nepal (6, 42), the prevalence of pre-diabetes 10.3% (95% CI: 6.1–14.4%) observed in this study further highlights a potential indicator of further progression of the epidemic in the country unless preventive measures are introduced at a large scale.

In this review, we found that the pooled prevalence of diabetes was 8.1% (95% CI: 7.3–8.9%) for urban populations and 1.03% (95% CI: 0.7–1.3%) for rural populations. A wealth of studies has reported that the problem of diabetes is largely concentrated in urban areas (43, 44). Few population-based studies conducted in Nepal so far have reported a high prevalence of type 2 diabetes in urban areas (21, 23). For instance, in a study by Singh et al. the prevalence of type 2 diabetes in an urban area of Nepal was 14.6% compared to 2.5% in a rural area (21). There is an increasing urbanisation in Nepal (45). The general shift of people from rural to urban areas for education, employment, and a better life may have contributed to an increasing burden of type 2 diabetes. It is also possible that people with diabetes may move to urban areas after diagnosis to be closer to hospitals, perhaps staying with urban family members.

This review found that being a woman is a significant risk factor for diabetes in Nepal. There is a paucity of Nepal-specific studies depicting high risk of type 2 diabetes among women; however, a systematic review from India reported that women are at higher risk of type 2 diabetes (46). The positive association we found between gender and type 2 diabetes has also been observed in Pakistan (47) and Turkey (48). One possible reason behind this might be due to low educational level of women as a result of which they might pay less attention to their dietary intake habits and physical activities. One statistic puts female literacy rate at around 47% as compared to 71% for men in Nepal (45). Moreover, owing to patriarchal mindset, women are normally expected to pay more attention to the health of the men and children in the family, and in the process they might ignore their own well-being (49). A wealth of studies has documented an association between diabetes and BMI (50), family history (51), physical inactivity (52), and area of residence (53). We could not perform a meta-analysis for these risk factors due to the limited number of studies in Nepal. More studies are needed to explore the association between diabetes and its risk factors in Nepalese settings.

Given the considerable burden of diabetes, there is a need for future research efforts focusing on preventive interventions and control measures in the Nepalese communities. It is necessary to consider the development of cost-effective intervention methods for diabetes prevention and control such as routine diabetes care including lifestyle counselling, early screening, monitoring of hyperglycaemia, provision of diabetes education, and self-management care programs (54, 55). At present, the government and few non-government organisations (NGOs) are conducting few awareness creation programs on the prevention of type 2 diabetes through health camps and by using the mass media in Nepal (56). However, diabetes and other NCDs are still not the priority area of the government and there is a paucity of programs to detect, manage, and prevent diabetes and NCDs in the country (57). Moreover, the level of knowledge, attitude, and good practice as a means to control and prevent diabetes is very low among Nepali people (20). Unless urgent and specific focus is on preventing, treating, and controlling of diabetes, the burden of diabetes will be severe in low-resource setting such as Nepal. Acquiring information regarding awareness level about diabetes is the first step in formulating prevention programs for diabetes (51). Therefore, a national strategy is required to address the disease burden and one of the strategies would be to involve a large number of community health care workers to communicate with the general public at large which can serve as an antecedent for future prevention and management efforts of type 2 diabetes in low-resource settings (58). Furthermore, information on the prevention and control of diabetes must be incorporated into general health promotion programs, from the government, NGOs and international non-government organisations (INGOs). Specific lifestyle interventions tailored to meet the cultural, religious, and socio-economic needs of the target communities are urgently needed.

### Limitations

This study has some limitations, which are noteworthy. Our study is limited only to the selected database source and English-language publications and therefore might have missed a small number of relevant publications. Considering the very low number of eligible articles, we took diabetes studies regardless of the place of study. We could not consider key variables that have shown to influence the prevalence of diabetes in this study such as BMI, family history, physical activity, and diet intake. More than two-thirds of studies included were of poor methodological quality in terms of sample size, variable selection, and sampling techniques, which might have resulted in some bias. Overall, a high degree of heterogeneity was observed in the included studies. Three articles fulfilled the criteria for reporting high-quality studies. The majority of the articles lacked the limitations of their studies. Two of the studies were hospital-based (19, 20), we considered removing those as they are not typical general population studies. However, the prevalence rates in those two studies (5.2 and 6.3%) were at the lower end of the range (one would expect a much higher prevalence in the hospital). These two hospital-based studies may have biased the urban–rural difference as hospitals in Nepal are based in urban areas but provide care for both urban and rural patients.

## Conclusions

Our pooled results support the finding that type 2 diabetes is currently a high-burden disease in Nepal suggesting a possible area for health promotion activities as well as early diabetes interventions to help control the disease. Further prevalence studies are needed with a robust method. In spite of the limited publications, our review suggests that there are considerable differences in the prevalence of type 2 diabetes between rural and urban areas and between studies. Consequently, there is a need to prioritise diabetes on the public health care agenda in Nepal through the promotion of preventive measures such as dietary pattern, exercises, and periodic check-up.
